# Productivity depends more on the rate than the frequency of N addition in a temperate grassland

**DOI:** 10.1038/srep12558

**Published:** 2015-07-28

**Authors:** Yunhai Zhang, Jinchao Feng, Forest Isbell, Xiaotao Lü, Xingguo Han

**Affiliations:** 1 State Key Laboratory of Vegetation and Environmental Change, Institute of Botany, Chinese Academy of Sciences, Beijing 100093, China; 2 State Key Laboratory of Forest and Soil Ecology, Institute of Applied Ecology, Chinese Academy of Sciences, Shenyang 110164, China; 3 Institute of Desertification Studies, Chinese Academy of Forestry, Beijing 100091, China; 4 Department of Ecology, Evolution and Behavior, University of Minnesota, St. Paul, MN 55108, USA

## Abstract

Nitrogen (N) is a key limiting resource for aboveground net primary productivity (ANPP) in diverse terrestrial ecosystems. The relative roles of the rate and frequency (additions yr^−1^) of N application in stimulating ANPP at both the community- and species-levels are largely unknown. By independently manipulating the rate and frequency of N input, with nine rates (from 0 to 50 g N m^−2^ year^−1^) crossed with two frequencies (twice year^−1^ or monthly) in a temperate steppe of northern China across 2008–2013, we found that N addition increased community ANPP, and had positive, negative, or neutral effects for individual species. There were similar ANPP responses at the community- or species-level when a particular annual amount of N was added either twice year^−1^ or monthly. The community ANPP was less sensitive to soil ammonium at lower frequency of N addition. ANPP responses to N addition were positively correlated with annual precipitation. Our results suggest that, over a five-year period, there will be similar ANPP responses to a given annual N input that occurs either frequently in small amounts, as from N deposition, or that occur infrequently in larger amounts, as from application of N fertilizers.

Nitrogen (N) is a major limiting resource for aboveground net primary productivity (ANPP) in diverse natural ecosystems^1^,^2^. As the world population continues to grow, more biologically available N is needed to increase ANPP for providing food, fuel, and fiber^3^. More reactive N is now fixed through anthropogenic production than via biological sources in terrestrial ecosystems^3^,^4^,^5^. In some developed countries, anthropogenic N production, and its undesirable side effects, such as N deposition, have been restricted and/or reduced during recent decades^3^,^6^, however, N deposition is expected to continue to increase worldwide in the coming decades^7^. In developing countries, N deposition is increasing, largely due to increased use of N fertilizer and increased combustion of fossil fuels^6^,^8^. For instance, total N deposition increased to as high as ~15 g N m^−2^ yr^−1^ in some areas of the North China Plain^9^. Global annual atmospheric N deposition is expected to continue to increase in coming decades^4^,^10^. As such, it is timely to accurately evaluate the sensitivity of ANPP in terrestrial ecosystems to increasing N deposition.

The frequency of N addition can determine the effects of N addition on ecosystem strucutre and functioning^11^,^12^. New experiments with a wide-range of rates and frequencies of N addition are needed to better understand how N deposition influences plant populations, communities, and ecosystems. In manipulated experiments aiming to explore the effects of increasing atmospheric N deposition on ecosystem functioning, reactive N is usually added in either dry or wet form to ecosystems during the growing season with only a few applications per year^13^,^14^,^15^. In contrast, atmospheric N deposition occurs as many frequent events, each of which adds a relatively small quantity of N to the ecosystem. Besides, atmospheric N deposition remains high over winter despite decreased agricultural activity because of increased fossil fuels consumption over this season. It has been reported that less N was available to plants in the spring when N was added during the previous winter than when N was added during the previous fall or early spring in a temperate grassland^16^. Thus, growing season and winter N additions should both be considered in new experiments. It has been hypothesized that results from previous experimental studies might lead to biased over-estimates of the effects of N deposition on communities and ecosystems, due to the excessively large and infrequent pulses of nutrients added to ecosystems in these studies^17^. However, the long-term effects of frequent N addition at multiple annual rates of N addition on ANPP have scarcely been tested in previous studies.

ANPP at the community-level generally shows a positive, but decelerating relationship with increasing N addition rates^14^,^15^,^18^, whilst species responses can be positive or negative^19^,^20^,^21^. ANPP could be affected by not only the annual rate but also by the within-year frequency of N addition. There is not yet concensus regarding how ANPP at the community- or species-level will respond to changes in the frequency of N addition. Previous studies have found that infrequent N inputs can lead to higher^22^,^23^,^24^, similar^25^,^26^, or lower^27^,^28^,^29^ plant producitivity than observed under frequent N inputs. Furthermore, individual species might respond positively or negatively to changes in the frequency of N inputs, depending in part on the correspondence between species’ phenonlogy and the timing of N inputs. For example, Bilbrough and Caldwell^23^ found higher ANPP of *Bromus tectorum* in low frequency of N addition because it was favored by early-spring N pulse in monoculture, whereas Yoder and Caldwell^26^ found that *B. tectorum* was not influenced by N addition frequency within multi-species mixtures. Few studies have considered the long-term effects of independent manipulations of both the annual rate and frequency of N addition on ANPP at both community- and species-levels.

Temperate steppe is a floristically diverse and productive grassland type of both ecological and economic importance in Eurasia^30^. There is increasing evidence that the temperate steppe is sensitive to global change^31^,^32^, particularly to N addition^11^,^14^,^33^. To assess the potential impacts of N deposition on this ecosystem, we designed an experiment that included nine rates of N addition as NH_4_NO_3_ (from 0 to 50 g N m^−2^ yr^−1^) crossed with two frequencies of N addition (twice or monthly yr^−1^) in a temperate steppe. To our knowledge, this is the first study that jointly considers the role of both the rate and frequency of N addition on ANPP over a relative long-term period (five years). We aimed to determine the response of ANPP, at both community- and species-levels, to different rates and frequencies of N addition. In the same experimental setup, both leaf chlorophyll content^34^ and soil ammonium concentration^11^,^12^ were higher at lower frequency of N addition, suggesting higher light capturing efficiency and higher available soil N for plant growth at lower frequency of N addition. Thus, based on these previous findings, and a previously proposed conceptual framework that plant growth might change slower at more frequent N addition^17^, we hypothesized that plant productivity would be higher at lower frequency of N addition. We also explored underlying mechanisms.

## Results

### Effects on ANPP at community-level

**Figure 1:**
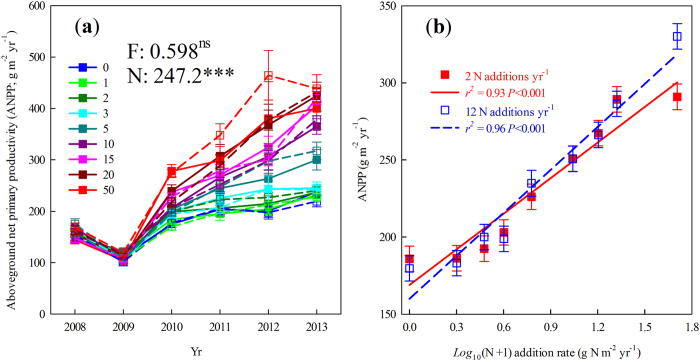
Temporal trend in aboveground net primary productivity (ANPP) at the community-level by N addition treatment. (**a**), Responses of community ANPP to experimental manipulations of N addition frequencies (filled symbols and solid lines = 2 N additions yr^−1^, open symbols and dashed lines = 12 N additions yr^−1^) and N-rate (different colors ranging from blue = low to red = high; g N m^−2^ yr^−1^). The values in 2008 showed the pre-treatment ANPP. *F*, N addition frequency and *N*, N-rate (*N* as the continuous factor), ^ns^ and ***: statistically significant at *P* > 0.05 and *P* < 0.001, respectively. (**b**), Result of repeated-measures analysis of variance for community ANPP. Regression parameters were estimated using log-linear model with N-rate as the continuous predictor, i.e., *ANPP* = *Intercept* + *Slope* × *log*_10_(N + 1). Error bars indicate 1 SE.

Community ANPP significantly increased with increasing N addition rates (Table 1; Fig. 1; *P* < 0.001). There was no significant effect of N addition frequency, and no interaction between N addition frequency and rate, on community ANPP (Table 1, Fig. 1). The effects of N addition rates on community ANPP varied among different years, as indicated by a significant interaction between N rates and year (Table 1; *P* < 0.001). Community ANPP increased with increasing N addition rates from 2010 to 2013, but not during the first year (2009) of the study (Fig. 1). Regardless of the N addition frequency, by the fifth year (2013), ANPP was higher with the addition of ≥5 g N m^−2^ yr^−1^. The response ratio of community ANPP following N addition was positively correlated with annual precipitation (August to the next July) (Fig. S1; *R*^*2*^ = 0.92, *P* < 0.001), similarly for both frequencies of N addition (Fig. S1; ANCOVA N addition frequency × annual precipitation interaction *F*_1,6_ = 0.23, *P* > 0.05).

### Effects on ANPP at species-level

**Figure 2:**
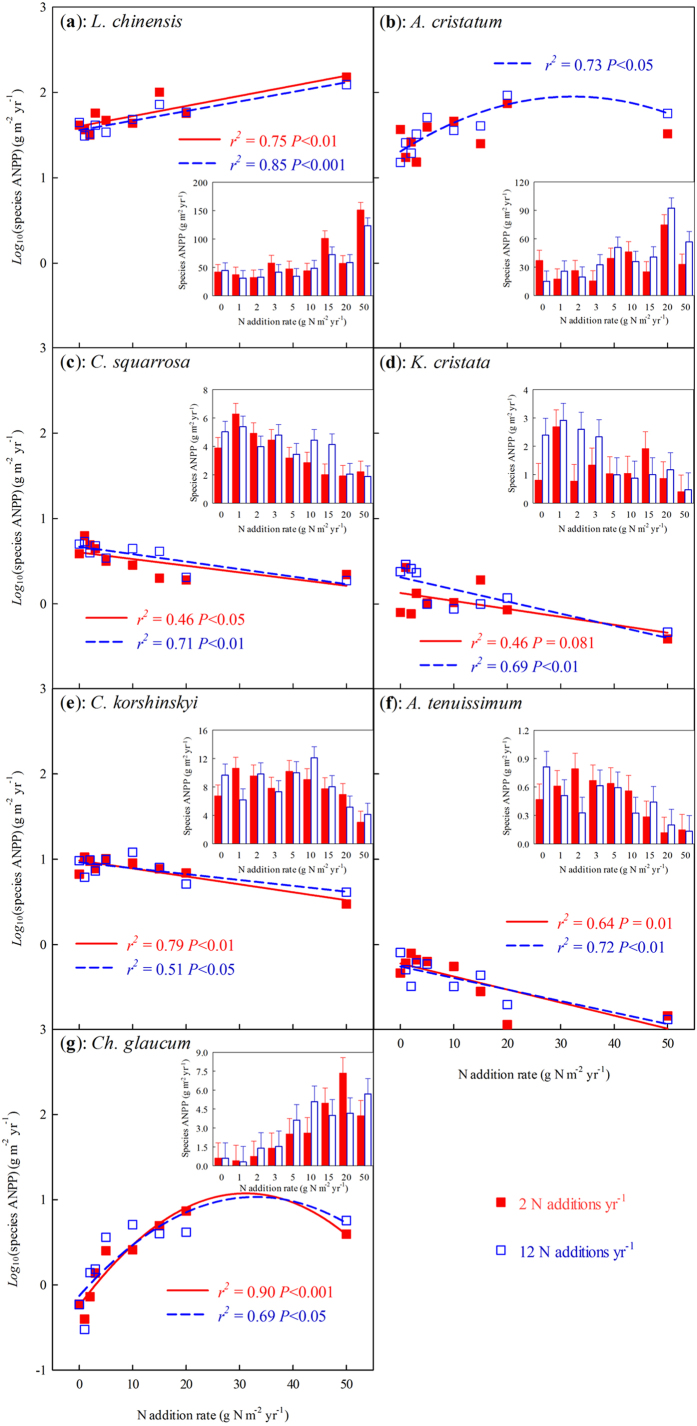
Effects of N addition on species ANPP across 2009–2013. (**a**), *L. chinensis*, (**b**), *A. cristatum*, (**c**), *C. squarrosa*, (**d**), *K. cristata*, (**e**), *C. korshinskyi*, (**f**), *A. tenuissimum*, and (**g**), *Ch. glaucum*, respectively. n = 10. The insets indicated the results of repeated-measures analysis of variance for species ANPP across 2009–2013. Error bars indicate 1 SE.

**Figure 3:**
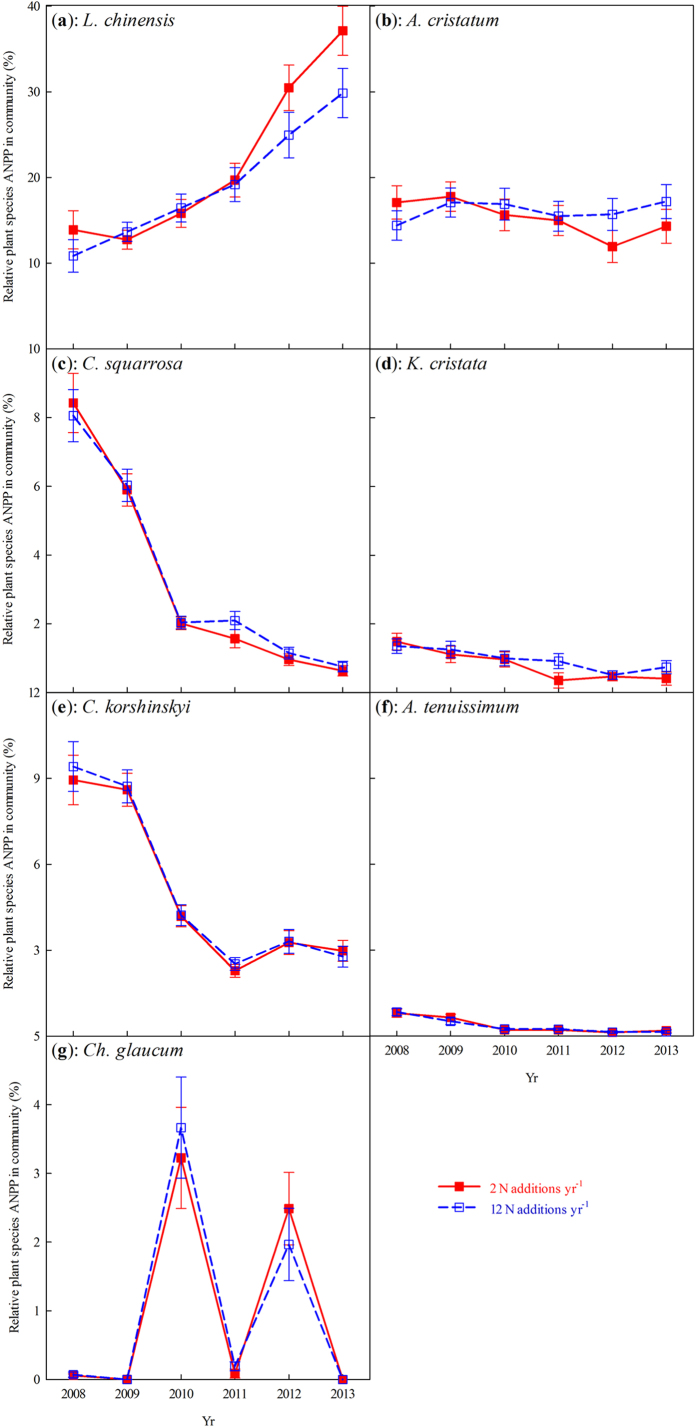
Temporal trends in effects of N addition on species relative ANPP in community across all N-rate. (**a**), *L. chinensis*, (**b**), *A. cristatum*, (**c**), *C. squarrosa*, (**d**), *K. cristata*, (**e**), *C. korshinskyi*, (**f**), *A. tenuissimum*, and (**g**), *Ch. glaucum*, respectively.

The ANPP of all species showed no detectable difference between the two frequencies of N addition (Table 1; Fig. 2). Nitrogen addition changed the ANPP of *L. chinensis*, *Agropyon cristatum*, *Cleistogenes squarrosa*, *Koeleria cristata*, *Carex korshinskyi*, *Allium tenuissimum*, and *Chenopodium glaucum*. Increasing the rate of N addition increased the ANPP of *L. chinensis* and *Ch. glaucum* and decreased that of *C. squarrosa*, *K. cristata*, *C. korshinskyi*, and *A. tenuissimum* at 2 N additions yr^−1^ (Fig. 2). Increasing the rate of N addition increased the ANPP of *A. cristatum* and decreased that of *K. cristata* at 12 N additions yr^−1^ (Table 1; Fig. 2). The ANPP of *L. chinensis*, *A. cristatum*, *C. squarrosa*, *C. korshinskyi*, and *Ch. glaucum* showed significant inter-annual variation. The effects of N additions on the ANPP of *L. chinensis*, *A. cristatum*, *C. korshinskyi*, and *Ch. glaucum* slowly manifested over time, as indicated by a significant interaction between year and the rate of N addition (Table 1). Neither one of the species relative ANPP in community was significantly different between the two frequencies of N addition across 2009–2013 (Fig. 3). Over time, N addition only increased the relative ANPP of *L. chinensis*, and decreased that of *C. squarrosa*, *K. cristata*, *C. korshinskyi*, and *A. tenuissimum* in this community (Fig. 3; *P* < 0.05). Nitrogen addition frequency and year interacted to affect the relative ANPP of *L. chinensis* (Fig. 3a; *F*_4,644_ = 2.4, *P* = 0.072). Nitrogen addition increased the relative ANPP of *L. chinensis* (Fig. 3a). In particular, at the fifth year (i.e. 2013), the relative ANPP of *L. chinensis* was greater in 2 than that in 12 N additions yr^−1^ (Fig. 3a; N addition frequency effect: *F*_1,161_ = 3.2, *P* = 0.075).

### Effects on soil conditions

**Figure 4:**
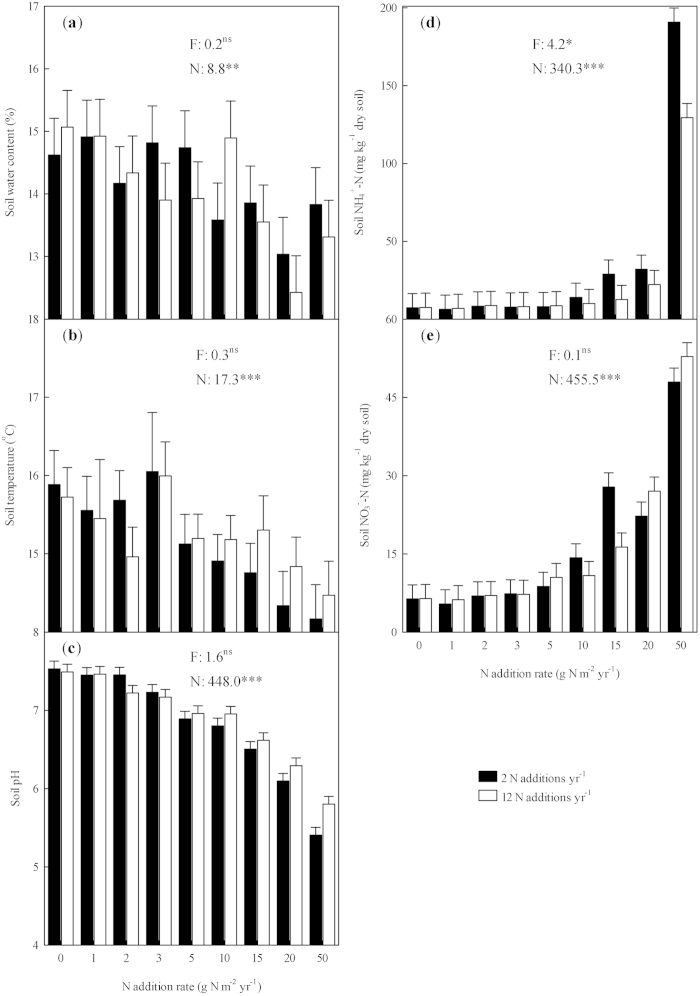
Effects of N addition on soil factors across 2012–2013. The top 10 cm soil water content (**a**), soil temperature (**b**), soil pH (**c**), soil ammonium concentration (NH_4_^+^–N); (**d**), and soil nitrate concentration (NO_3_^−^–N); (**e**) in response to the two N addition frequencies (filled symbols = 2 N additions yr^−1^, open symbols = 12 N additions yr^−1^) and N-rate. Error bars indicate 1 SE. *F*, N addition frequency and *N*, N-rate (*N* as the continuous factor), ^ns^, *, **, and ***: statistically significant at *P* > 0.05, *P* < 0.05, 0.01, and 0.001, respectively.

Increasing the rate of N addition decreased the top 10 cm soil water content, soil temperature, and soil pH, and significantly increased soil NH_4_^+^–N and NO_3_^−^–N (Fig. 4; All *P* < 0.05). In contrast, decreasing the frequency of N addition significantly increased soil NH_4_^+^–N (Fig. 4d; *P* < 0.05), but had no significant effect on the other four soil variables^11^.

### Relationships between changes in ANPP at community-level and soil conditions

**Figure 5:**
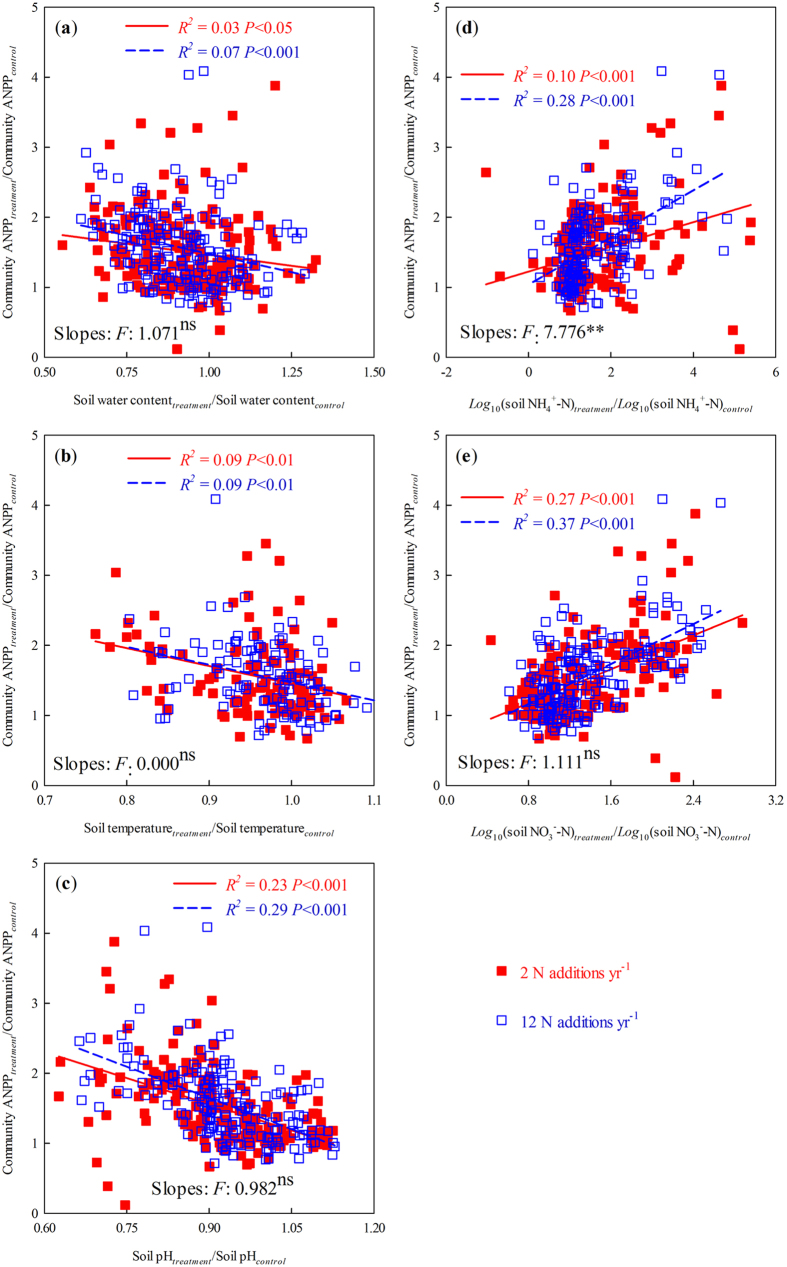
Relationships between changes in the top 10 cm soil characteristics and community ANPP across 2012–2013. Soil characteristics , (**a**), soil water content, (**b**), soil temperature, (**c**), soil pH, (**d**), soil NH_4_^+^–N, and (**e**), soil NO_3_^−^–N, respectively. In **d** and **e**, soil NH_4_^+^–N and soil NO_3_^−^–N were *log*_10_ transformed to meet the normality and homogeneity. *F*-ratio was given for the result of analysis of covariance of the two slopes in each subplot. ^ns^ and **: statistically significant at *P* > 0.05 and *P* < 0.01, respectively.

In 2012 and 2013, regardless of the frequency of N addition, the community ANPP response to N addition was significantly negatively correlated with soil water content (Fig. 5a; *P* < 0.05), soil temperature (Fig. 5b; *P* < 0.05), and soil pH (Fig. 5c; *P* < 0.001), and had a positive relationship with *log*_10_(soil NH_4_^+^–N) (Fig. 5d; *P* < 0.001) and *log*_*10*_(soil NO_3_^−^–N) (Fig. 5e; *P* < 0.001). The slope of community ANPP responding to *log*_10_(soil NH_4_^+^–N) was greater under 12 than under 2 N additions yr^−1^ (Fig. 5d; N addition frequency effect: *F*_1,356_ = 7.776, *P* < 0.01). There were no apparent difference between 2 and 12 N additions yr^−1^ for the slopes of community ANPP response to the other four soil factors (Fig. 5).

## Discussion

Consistent with results from previous studies in this ecosystem^14^,^19^ and other grasslands^18^,^35^, we found that ANPP at the community-level showed a positive and decelerating relationship with increasing N addition rates. The impacts of N addition rates on community ANPP are relatively well studied. Together, these studies indicate that N is often a limiting factor in global grasslands^1^.

Contrary to our initial hypothesis, we found no detectable impacts of changing the frequency of N addition on ANPP at the community- or species-level. To date, our study might be one of the best-designed tests of the effects of the frequency of N addition on ecosystem functioning (e.g. ANPP) across multiple years. This suggests that plant ANPP will respond similarly to a given annual rate of N inputs, regardless of whether N is added in a few large fertilization events or in many small deposition events in temperate grassland where air temperature peaks with rainfall^36^. Previous studies considering the effects of the frequency of N addition on ANPP have rarely been conducted for longer than two growing seasons, have rarely considered responses at both the community- and species-levels, and have produced mixed results.

At the community-level, previous studies might provide reliable estimates for the short-term impacts of N deposition on community ANPP, despite the fact that they have not applied N in small and frequent pulses that most closely resemble N deposition. Within two-year treatments, Barton and colleagues^22^ found that the ANPP of *Pennisetum clandestinum* turfgrass community was greater at low than high frequency of N addition in Australia where air temperature peaks with rainfall. In contrast, community ANPP, within one growing season treatment, was 34% greater at high than low frequency of N addition in a Kansas prairie, US where air temperature peaks with rainfall^28^. Although we found significantly lower community species richness at lower frequency of N addition^11^, no significant difference was found in community ANPP in the same experimental setup. The lost species contributed little to community ANPP^11^, which likely partly explains why diversity, but not productivity, responded to the frequency of N addition.

At the species-level, both Bilbrough and Caldwell^23^ and Campbell and Grime^24^ found that the ANPP of all experimental species was greater at low than high frequency of N addition in greenhouse where air temperature peaks with water addition. In contrast, other studies found that the ANPP of grasses, *Calamagrostis purpurascens*, *Deschampsia caespitosa* and *Trisetum spicatum*^27^ and *Schismus arabicus*^29^ were greater at high than low frequency of N addition. The frequency of N addition has no significant effect on the ANPP of some species, such as *Carex rupestris*, *Kobresia myosuroides* and *C. scopulorum* at Niwot Ridge, Colorado^27^, *B. tectorum* at Sanpete County, Utah^26^ and *Panicum antidotale* at Cambridge^25^. These mixed results in previous studies under different frequencies of N addition might be partly due to the presence or absence of species interactions^26^,^37^. All the studies that have controlled the frequency of N addition are carried out in ecosystems with a similar climate as our present location, in that air temperature peaks with rainfall. In our study, seven species were influenced by the rate of N addition, but no species were influenced by the frequency of N addition (Fig. 2). *Ch. glaucum* is an annual species and the other six response species are perennial species^11^. Perennial species could store nutrients in their roots for plant growth during the following growing season. Thus, we suspect that many perennial species were unaffected by the frequency of N addition because they integrated the added N and used it over a long time horizon, regardless of whether it arrived in a few large pulses or many smaller pulses. For the annual species, we suspect that competition for other resources (such as light, soil water, etc.) with neighboring perennial clone species, such as *S. grandis* and *L. chinensis*, resulting the similar ANPP of *Ch. glaucum* between the different frequencies of N addition. Thus, both species life forms and species interactions likely influenced species’ responses to N addition.

In addition to the increase in N availability, acidification of the soil is one of the main effects of N enrichment^38^,^39^,^40^, which results in the loss of acid and ammonium sensitive species^41^. It has been reported that higher cumulative soil NH_4_^+^–N under less frequent N addition treatments can result in less recruitment, slower plant growth at both the juvenile and adult stages and more rapid species loss^42^,^43^,^44^. We also found decreases in both soil water content and soil temperature with increases in ANPP at the community-level after N addition. The sharp depletion of soil water by the enhancement of ANPP after N enrichment suggests that the plant community could become high-yielding to water increases under N enrichment, and may partly explain why the ANPP response to N was positively correlated with annual precipitation. Acidification and reduction of soil water and temperature often have negative effects on plant species diversity by suppressing seed germination and inhibiting seedling establishment in temperate grassland^41^,^45^. Given the increases in ANPP at the community-level by N enrichment, there could be tradeoffs between N-induced increases in productivity and losses of biodiversity for land N-related management strategies and policies^14^,^15^.

Annual precipitation is often the primary driver for ANPP in semi-arid regions^46^,^47^. We found that there was no effect of N addition at any rate during the driest year (i.e. 2009) and that increases in annual precipitation sharply increased community ANPP responses to N addition (Fig. S1). Hooper and Johnson^48^ proposed that a significant increase of community ANPP with increasing in annual precipitation indicates community is co-limited by water and N. In our study region, ANPP significantly increase with water addition, regardless of whether N is added^49^. Our results are consistent with results from previous studies, which showed that the combination of both water and N additions often have an additive effect on ANPP in semi-arid ecosystems^48^,^49^,^50^. Significant increases of annual precipitation^51^ and N deposition^10^ are predicted in North China in the following decades. Our study ecosystem is conservative in nutrient use with a slow decomposition rate and a high nutrient retention rate under N addition^52^. Together, these results suggest that future changes in precipitation and N deposition could result in increases in ANPP and carbon sequestration, which could help reduce the build-up of greenhouse gases in the atmosphere.

In summary, our study revealed that ANPP responses to increasing rates of N addition at both the community- and species-levels were independent of the frequency of N addition over a five-year time horizon in temperate grassland where air temperature peaks with rainfall events. We found no evidence for the hypothesis that lower N addition frequency would lead to higher ANPP, but the community ANPP was less sensitive to soil ammonium at low frequency of N addition. We also found that the enhancement of productivity by N addition is largely dependent on annual precipitation, highlighting the important role of water availability in mediating ecosystem responses to N deposition in temperate steppe. From a perspective of grassland management and hay-making, combining infrequent N addition with irrigation might help improve productivity in Eurasian grassland, and minimize the costs of N application. Further study over a longer time horizon will be needed to determine whether accumulation of soil ammonium and changes in species dominance will eventually lead to significant differences in community ANPP under different N frequency treatments. More new long-term experiments that independently manipulate the rate and frequency of N addition are needed to examine the generality of our results in other ecosystems, for example Mediterranean regions where rainfall does not peak with temperature. Conservation practitioners, land managers, and policy-makers may need to take into account the trade-off between the increases in productivity and losses of biodiversity under N fertilization or deposition.

## Methods

### Study site

The field experiment was carried out in a temperate steppe near the Inner Mongolia Grassland Ecosystem Research Station (IMGERS; 116°14′E, 43°13′N), which is located in the Xilin River Basin, Inner Mongolia Autonomous Region, China. The 50 ha field has been fenced since 1999 to exclude large animal grazing. The topography of the experimental area is flat, with an elevation range of 1255–1260 m. The mean annual temperature was 0.9 °C, with mean monthly temperatures ranging from –21.4 °C in January to 19.7 °C in July for the period of 1980–2013. Mean annual precipitation was 351.4 mm, with approximately 72.8% falling during the growing season from May to August. The soil is classified as Haplic Calcisols and Calcic-Orthic Aridisol by the FAO and the US soil classification system, respectively. The plant community was dominated by two species, *Stipa grandis* P. Smirn and *Leymus chinensis* (Trin.) Tzvel, which together accounted for >60% of the total peak aboveground biomass (Table 2). There were no legumes at our experimental site. This site received no fertilizer prior to this experiment. The ambient total (wet and dry) N deposition in this region was less than 1.5 g N m^−2^ yr^−1^ for recent two decades^53^.

### Experimental design

The experiment was established during September 2008, following a randomized complete block design^12^. There are nine rates (0, 1, 2, 3, 5, 10, 15, 20, and 50 g N m^–2^ yr^–1^) crossed with two frequencies of N addition (2 times vs. 12 times N addition yr^−1^). There is also an unamended control treatment (control, with no additions of N, water, or sand) to detect the influence of water and sand additions (see below paragraph) by comparing responses between the control and 0 N addition plots. Each plot is 8 × 8 m. Hence, there were 19 experimental treatments in total, all present in each of ten blocks.

Purified NH_4_NO_3_ (>99%) addition started on 1 September 2008 and continued on the first day of each month thereafter for the high frequency treatment (12 N additions yr^−1^), and started on 1 November 2008 and continued on the first day of June and November thereafter for the low frequency treatment (2 N additions yr^−1^). That is, when the monthly N addition in August per year, it is with total annual equal N loadings between 2 and 12 N additions yr^−1^. During the growing season from May to October, fertilizer was weighed and mixed with purified water (9.0 L total for all treatments receiving water: either 9.0 L once in June or 1.5 L monthly from May to October), and sprinkled evenly using a sprayer to each plot to simulate wet N deposition. It was estimated that less than 1 mm of water was added to each plot annually, except the control plots, which had no water added. In winter (from November to April), NH_4_NO_3_ was mixed with sand (because of low amount of added NH_4_NO_3_ at low N rates in 12 N additions yr^−1^; 0.5 kg total for each treatment receiving sand: either 0.5 kg once in November or 0.08 kg monthly from November to April) and broadcast uniformly by hand. Sand was sieved through less than 1 mm in size, hydrochloric acid dipped, washed in purified water, and then heated at 120 °C for 24 hours in an oven. To avoid otherwise potentially confounding effects, plots received the same amount of water and sand, regardless of whether they received the high or low frequency of N addition treatment.

### Field sampling and measurements

ANPP was estimated annually from peak aboveground biomass. Peak aboveground biomass is an acceptable approximation for ANPP in this region as aboveground plant tissues die during the winter season^14^. Plant aboveground biomass was sampled each year from 2008–2013 between 10 and 15 August using a 0.5 m × 2 m quadrat, which was randomly placed in each plot without spatial overlap of quadrats among years and at least 50 cm inside the border of each plot to avoid edge effects. All living vascular plants were sorted to species, oven-dried at 65 °C for 48 hours to a constant weight, and then weighed.

Concurrent with aboveground plant sampling in 2012 and 2013, three soil cores (0–10 cm depth and 50 cm apart) were collected using a 3 cm diameter soil auger adjacent to each aboveground plant sampling plot. The soil samples were thoroughly mixed and sieved through a 2 mm mesh to obtain one composite sample for laboratory analysis of soil ammonium (NH_4_^+^–N; mg kg^−1^) and nitrate (NO_3_^−^–N; mg kg^−1^) concentration and soil water content (%). Subsamples were air-dried for analysis of soil pH. For soil NH_4_^+^–N and NO_3_^−^–N measurements, 10 g fresh soil subsamples were extracted with 50 ml 2.0 M KCl solution and then analyzed using a flow injection auto analyzer (FLAstar 5000 Analyzer; Foss Tecator, Hillerød, Denmark). The soil NH_4_^+^–N and NO_3_^−^–N concentration is expressed on a dry weight basis. The soil pH was measured in water suspension (soil:water = 1:2.5) by a pH meter (FE20–FiveEasy). Soil temperature (°C) was automatically recorded within each plot at a 1.5 hour interval by iButton digital temperature loggers (DS1922L) at a depth of 10 cm in the soil beginning in April 2012. We used the average temperature of growing seasons (from May to 9 August) for further analyses in both 2012 and 2013.

### Statistical analyses

Repeated-measures analysis of covariance (ANCOVA) were used to test effects of N addition frequency, N–rate (N as a continuous factor), year, and their interactions on the ANPP of the community and of species and species relative ANPP, using the data in 2008 (pre-treatment measurement) as a covariate to control for initial differences in ANPP across plots (Type III SS; α = 0.05). The species relative ANPP was calculated as, 
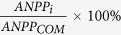
, where *ANPP*_*i*_ is the *i* species ANPP in the community, and *ANPP*_*COM*_ is the community ANPP. Repeated-measures analysis of variance (ANOVA) were employed to test the effects of N addition frequency, N–rate (N as a continuous factor), year, and their interactions on the top 10 cm soil water content, temperature, pH, NH_4_^+^–N, and NO_3_^−^–N concentration across 2012–2013. The response ratio of ANPP, soil water content, soil temperature, soil pH, soil NH_4_^+^–N, and NO_3_^−^–N concentration was calculated as the ratio of these values in treatments divided by the mean in controls, ANPP for example, ANPP_*treatment*_/ANPP_*control*_. The response ratio of soil NH_4_^+^–N and NO_3_^−^–N were *log*_10_ transformed to meet the assumptions of normality and homogeneity. ANCOVA was employed to distinguish the slopes between the two N addition frequencies when ANPP responded to each environmental factor (annual precipitation, soil water content, temperature, pH, NH_4_^+^–N, and NO_3_^−^–N) at the community-level. All statistical analyses were performed with the SPSS software package (SPSS 17.0 for windows, SPSS Inc., Chicago, IL, USA).

## Additional Information

**How to cite this article**: Zhang, Y. *et al.* Productivity depends more on the rate than the frequency of N addition in a temperate grassland. *Sci. Rep.* **5**, 12558; doi: 10.1038/srep12558 (2015).

## Supplementary Material

Supplementary Information

## Figures and Tables

**Table 1:** Results of repeated-measures analysis of covariance (ANCOVA) testing the effects of the frequency and rate of N addition on aboveground net primary productivity (ANPP) for community (*COM*) and species, *L.c.*, *Leymus chinensis*, *A.c.*, *Agropyon cristatum*, *C.s.*, *Cleistogenes squarrosa*, *K.c.*, *Koeleria cristata*, *C.k.*, *Carex korshinskyi*, *A.t.*, *Allium tenuissimum*, and *Ch.g.*, *Chenopodium glaucum*, using the N-rate as the continuous variable and the initial value in 2008 as a covariate (V_0_) across 2009–2013.

	**df**	**COM**	**L.c.**	**A.c.**	**C.s.**	**K.c.**	**C.k.**	**A.t.**	**Ch.g.**
V_0_	1,176	0.2	21.4***	13.9***	63.1***	131.4***	10.7**	19.3***	0.3
N-rate (N)	1,176	247.2***	85.6***	9.2**	26.3***	20.0***	10.2**	17.5***	20.9***
N frequency (F)	1,176	0.6	1.9	1.2	1.1	0.0	2.3	0.2	0.1
Year (Y)	4,704	7.0***	12.0***	4.8**	3.7*	6.1***	1.4	0.6	8.5***
Y × N	4,704	40.5***	48.8***	2.5	1.5	2.8*	0.6	0.4	8.0***
Y × F	4,704	0.7	1.5	1.1	0.9	0.3	1.3	0.4	0.1
Regression parameters¶									
*Slope*	2 N	76.0***	55.0*	13.3	−2.2*	−0.4	−2.4	−0.3*	3.5**
12 N	92.9***	42.8*	32.6*	−1.9**	−1.5**	−2.1	−0.3**	3.4***	

Asterisks denote significant levels: *P < 0.05; **P < 0.01; and ***P < 0.001, respectively.

^¶^Regression parameters were estimated using log-linear model with N-rate as the continuous predictor, i.e., *ANPP* = *Intercept* + *Slope* × *log*_10_(N + 1). 2 N = 2 N additions yr^−1^ and 12 N = 12 N additions yr^−1^, respectively.

**Table 2:** Major species of plant functional groups within the quadrats (0.5 m×2 m) in control across 2009–2013

**Functional groups**	**Scientific name**	**Family**	**Height (cm)**	**Relative aboveground productivity in community (%)**	**Frequency**	**Frequency rank (from low to large)**∗
Dominant	*Stipa grandis*	Gramineae	69.05	35.60	1.00	E
	*Leymus chinensis*	Gramineae	45.70	25.22	0.87	E
Subdominant	*Achnatherum sibiricum*	Gramineae	58.82	16.49	0.90	E
	*Agropyon cristatum*	Gramineae	42.61	11.64	0.88	E
	*Carex korshinskyi*	Cyperaceae	20.37	4.55	0.97	E
	*Cleistogenes squarrosa*	Gramineae	19.21	3.29	0.69	D
	*Koeleria cristata*	Gramineae	22.13	1.01	0.44	C
Rare	*Poa subfastigiata*	Gramineae	56.19	0.65	0.23	B
	*Festuca dahurica*	Gramineae	32.88	0.12	0.06	A
	*Allium tenuissimum*	Liliaceae	23.50	0.40	0.40	C
	*Iris tenuifolia*	Iridaceae	19.56	0.10	0.07	A
	*Allium bidentatum*	Liliaceae	20.75	0.06	0.07	A
	*Allium ramosum*	Liliaceae	17.50	0.06	0.13	A
	*Thalictrum petaloideum*	Ranunculaceae	37.75	0.04	0.02	A
	*Potentilla bifurca*	Rosaceae	22.80	0.03	0.05	A
	*Chenopodium glaucum*	Chenopodiaceae	36.60	0.25	0.23	B
	*Axyria amaranthoides*	Chenopodiaceae	8.00	0.13	0.14	A
	*Salsola collina*	Chenopodiaceae	14.00	0.12	0.13	A
	*Artemisia scoparia*	Compositae	40.00	0.05	0.05	A
	*Saussurea japonica*	Compositae	11.67	0.05	0.02	A
	*Dontostemon micranthus*	Cruciferae	14.00	0.04	0.04	A
	*Chenopodium aristatum*	Chenopodiaceae	9.05	0.01	0.07	A

^∗^Species frequency rank classification: A, 0.01–0.20, B, 0.21–0.40, C, 0.41–0.60, D, 0.61–0.80, and E, 0.81–1.00, respectively.
