# Supplementary material for: Productivity depends more on the rate than the frequency of N addition in a temperate grassland
Source: Sci Rep. 2015 Jul 28;5:12558. doi: 10.1038/srep12558 (PMC4517389; doi:10.1038/srep12558)
Supplement: Supplementary Information [file srep12558-s1.pdf]

Title: Productivity depends more on the rate than the frequency of N addition in a temperate grassland

Yunhai Zhang[1,]*, Jinchao Feng[1,3], Forest Isbell[4], Xiaotao Lü[2,]*, Xingguo Han[1,2,]*

[1]State Key Laboratory of Vegetation and Environmental Change, Institute of Botany, Chinese Academy of Sciences, Beijing 100093, China

[2]State Key Laboratory of Forest and Soil Ecology, Institute of Applied Ecology, Chinese Academy of Sciences, Shenyang 110164, China

[3]University of Chinese Academy of Sciences, Yuquan Road, Beijing 100049, China

[4]Department of Ecology, Evolution and Behavior, University of Minnesota, St. Paul, MN 55108, USA

*Correspondence: zhangyh670@ibcas.ac.cn (Yunhai Zhang), lvxiaotao@iae.ac.cn (Xiaotao Lü) or xghan@ibcas.ac.cn (Xingguo Han). Tel.: +86 24 83970301, Fax +86 24 83970300

Supporting Information:

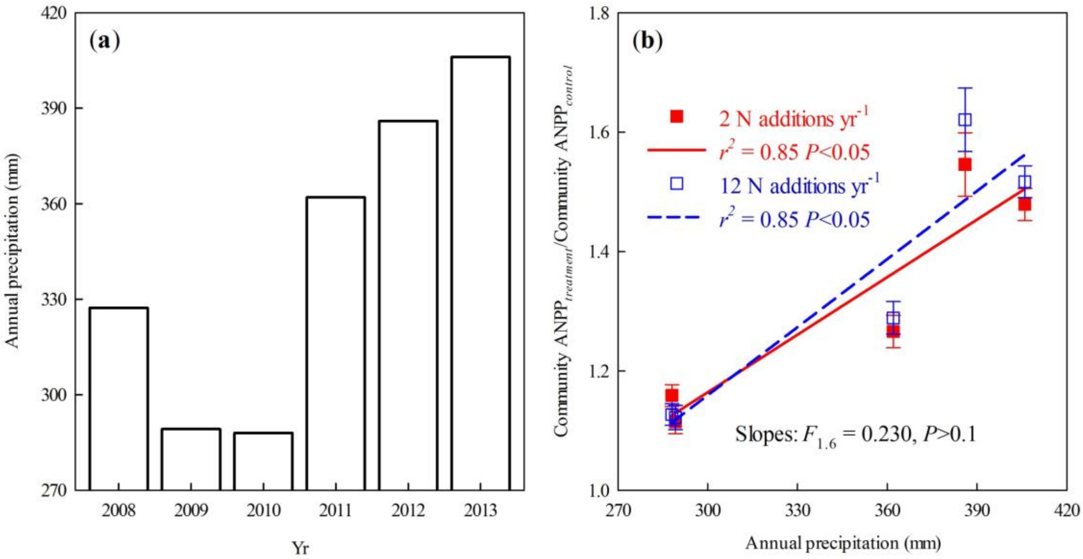

Figure S1 Annual precipitation (from August to next July) and correlation with the changes in community aboveground net primary productivity (ANPP) across 2009–2013. n = 80. Error bars indicate 1 SE. F-ratio was given for the result of analysis of covariance of the two slopes ($F_{1,6}$ = 0.230, P>0.1), so the two slopes showed no difference.
